# Symptom-Triggered Alcohol Detoxification Compared to Fixed-Dose Regimen of Benzodiazepines: A Retrospective Case–Control Study

**DOI:** 10.3390/brainsci15070758

**Published:** 2025-07-17

**Authors:** Laurent Becciolini, Fabienne Wehrli, Jens Kronschnabel, Carolina Wiesendanger, Norbert Scherbaum, Patrik Roser

**Affiliations:** 1 Center for Addictive Disorders, Department of Adult Psychiatry and Psychotherapy, University Hospital of Psychiatry Zurich, University of Zurich, 8001 Zurich, Switzerland; laurent.becciolini@pukzh.ch; 2 Institute of Psychology, Department of Clinical Psychology, University of Zurich, 8006 Zurich, Switzerland; fabienne.wehrli@psychologie.uzh.ch; 3 Department of Psychiatry and Psychotherapy, Psychiatric Services Aargau, 5210 Windisch, Switzerland; jens.kronschnabel@pdag.ch (J.K.); carolina.wiesendanger@bts-glarus.ch (C.W.); 4 Department of Addictive Behavior and Addiction Medicine, and Department of Psychiatry and Psychotherapy, LVR University Hospital Essen, Medical Faculty, University of Duisburg-Essen, 45147 Essen, Germany; norbert.scherbaum@uni-due.de

**Keywords:** alcohol detoxification, withdrawal symptoms, symptom-triggered therapy, Hamburg Alcohol Withdrawal Scale, fixed-dose regimen, benzodiazepine

## Abstract

Background: Alcohol withdrawal syndrome is a common clinical challenge that may lead to significant complications if not properly managed. Symptom-triggered therapy (STT) represents a promising alternative to fixed-dose regimens (FDRs) providing benzodiazepine prescriptions based on objectively quantified withdrawal symptoms. This study aimed to evaluate the effectiveness and safety of STT using the Hamburg Alcohol Withdrawal Scale (HAES) compared to FDRs in the management of inpatient alcohol detoxification. Methods: In a retrospective case–control study, alcohol detoxification treatment in STT was compared with FDRs. During a twelve-month observation period, a total of 123 patients in the STT group were recruited and compared with 123 controls in the FDR group (matched according to sex, age, and current amount of alcohol consumption) treated in the same hospital before the implementation of STT. The study outcomes included the total benzodiazepine dosage, duration of acute detoxification phase, length of inpatient stay, and occurrence of complications such as epileptic seizures and delirium tremens. Results: STT showed a significantly lower total benzodiazepine dosage (22.50 mg vs. 115.00 mg, *p* < 0.001), a shorter duration of the detoxification phase (48.00 h vs. 201.75 h, *p* < 0.001), and a reduced length of inpatient stay (23.00 days vs. 28.00 days, *p* = 0.003) compared to FDRs. There were no significant differences in the rates of complications between the two settings. Linear mixed model analysis revealed that the differences remained highly significant even after adjusting for various explanatory variables (i.e., age, sex, standard units of alcohol, psychiatric comorbidities, treatment discontinuation, and occurrence of any complication). Conclusions: STT appears to be as effective and safe as traditional fixed-dose regimens of benzodiazepines for the management of inpatient alcohol detoxification. This approach may thereby minimize unnecessary pharmacological exposure, facilitate the earlier integration of patients into psychoeducational and psychosocial interventions, and reduce healthcare costs.

## 1. Introduction

Alcohol use disorder (AUD) is one of the most disabling chronic diseases that significantly contributes to the global burden of disease, especially in Western countries [1]. In Switzerland, chronic hazardous alcohol use affects about 4.4% of all men and 3.4% of all women aged 15 years and older [2], and between 250,000 and 300,000 persons suffer from alcohol dependence [3]. Every year, about 25,500 persons require inpatient treatment due to alcohol intoxication or alcohol dependence [4], and about 1600 deaths per year are attributable to alcohol consumption [5]. Moreover, AUD produces tremendous social costs of about CHF 2.8 billion per year [6].

Prolonged excessive alcohol consumption is typically associated with compensatory neurochemical adaptations, including reduced GABAergic inhibitory transmission and increased glutamatergic excitatory transmission [7]. The abrupt cessation of alcohol can produce an alcohol withdrawal syndrome (AWS) as a result of unopposed neuroadaptation, leading to neurochemical imbalances and excessive neuronal activity. Approximately 50% of patients with AUD experience at least mild withdrawal symptoms upon cessation, such as anxiety, tremors, diaphoresis, hypertension, and tachycardia, and up to 5% may develop serious complications such as epileptic seizures and delirium tremens [8].

The standard treatment to reduce acute withdrawal symptoms and to prevent complications involves the use of benzodiazepines, such as diazepam, which enhance GABAergic inhibitory transmission via the positive allosteric modulation of GABA_A_ receptors and thereby attenuate neuronal hyperexcitability [7]. They are usually administered on a predetermined fixed-dose regimen (FDR), although pharmacological intervention is not required in many cases [9]. Therefore, patients with mild AWS may face overtreatment and receive more medication than necessary, leading to various side effects and an increased risk of developing benzodiazepine dependence [10]. On the other hand, patients with severe AWS may be undertreated and receive less medication than needed, thereby increasing the risk of serious complications and premature discontinuation of treatment [11]. In both cases, a fixed-dose regimen not only poses health risks but may also contribute to a prolonged acute withdrawal phase and increased length of inpatient stay [11]. In Switzerland, the latter aspect is of particular relevance since a new remuneration system (TARPSY) with decreasing daily rates for the reimbursement of inpatient psychiatric care was introduced in 2018, thereby providing financial incentives for reducing hospital length of stay [12].

Symptom-triggered therapy (STT) represents a promising alternative to FDRs, providing medication with benzodiazepine based on objectively quantified withdrawal symptoms [9]. This approach requires frequent and accurate monitoring by employing a validated withdrawal scale, such as the Clinical Institute Withdrawal Assessment for Alcohol-Revised (CIWA-Ar) scale [13]. Only four retrospective cohort studies have found that STT protocols significantly reduced benzodiazepine dosage, the duration of acute detoxification, and the total length of stay compared to FDRs, whereas the rate of complications and premature discontinuation of treatment did not differ between the two settings [14,15,16,17]. However, a meta-analysis of randomized controlled trials reported inconclusive evidence regarding mortality and complications and only moderate-strength evidence regarding withdrawal medication and treatment duration [18]. While this might be due to relatively small sample sizes and the inclusion of low-risk study populations with up to 60% of patients who did not require any pharmacotherapy [19], another study reported increased mortality and lengths of stay after the introduction of an STT protocol for AWS management [20].

Although introduced more than 30 years ago and, meanwhile, strongly recommended by national and international guidelines [21], STT has still been too little put into routine clinical practice, both internationally and in Switzerland [15]. One reason might be that some of the scales include too many criteria, making them more complicated and time-consuming, or do not consider important clinical variables such as blood pressure, heart rate, and/or previous serious complications [22]. Over the years, a variety of adaptations of the CIWA-Ar scale have been developed, including the Hamburg Alcohol Withdrawal Scale (HAES) [23]. The HAES differs from other scales in its simplicity and tabular organization, which facilitates the quick assessment of the severity and progression of AWS. It consists of only six items, which also comprise a history of delirium and seizures as the strongest predictor for future similar events [24]. Another advantage of the HAES is its validation for the prescription of clomethiazole, as well as oxazepam, which represents a reasonable alternative to diazepam due to its shorter half-life, thereby reducing the risk of accumulation and potential sedation; its lack of active metabolites; and its lower risk of drug–drug interactions [25].

The aim of this study was to evaluate the effectiveness of STT by using the HAES compared to FDRs in a clinical setting at a specialized treatment center for addictive disorders. Therefore, the electronic medical records of patients who underwent inpatient alcohol detoxification before and after the implementation of STT were analyzed. It was hypothesized that STT is associated with (1) the reduced dosage of benzodiazepines and (2) the shorter duration of the acute detoxification phase compared to FDRs, whereas (3) the rate of complications, transfers to a somatic hospital, and the premature discontinuation of treatment do not differ from FDRs.

## 2. Materials and Methods

### 2.1. Study Setting

This study was conducted at the Center for Addictive Disorders of Psychiatric Services Aargau (PDAG, Windisch, Switzerland). The PDAG ensures inpatient psychiatric treatment for the approximately 680,000 inhabitants of the Canton of Aargau, Switzerland. The Center for Addictive Disorders runs three specialized wards with a total of 58 inpatient treatment places and provides integrated multidisciplinary treatment for patients with substance use disorders, including qualified alcohol withdrawal treatment.

### 2.2. Study Procedure

For this case–control study, adult patients with a diagnosis of AUD according to the ICD-10 diagnostic criteria (F10) who were admitted to the Center for Addictive Disorders for inpatient alcohol withdrawal treatment were evaluated for study eligibility. Exclusion criteria were comorbid substance use disorders (F11–F19) except nicotine (F17) and completion of alcohol detoxification prior to admission in order to minimize confounders. In particular, comorbid substance use disorders may have a significant impact on the severity and progression of AWS but may also be associated with overlapping withdrawal symptoms caused by the cessation of additional substances. Eligible patients were divided into two groups according to the type of treatment. The patients treated with FDRs for alcohol detoxification were typically administered with diazepam starting with 5 mg four times per day or oxazepam starting with 15 mg four times per day when the breath alcohol concentration (BAC) fell below 1.0‰ and withdrawal symptoms had developed. The dosage was gradually reduced over a time period of seven to ten days, according to a daily clinical evaluation [10]. In July 2018, the FDR was replaced by STT with oxazepam using the HAES protocol as the new standard procedure across all wards [23]. In order to avoid bias due to the implementation and standardized training phase, the STT group consisted of patients who were admitted in the period between January and December 2019. In the next step, STT patients were carefully matched to pre-implementation-phase patients who underwent inpatient alcohol withdrawal treatment between July 2017 and June 2018 and who were treated with FDRs. No other major changes in treatment protocol or staff occurred between the two periods. The matching was based on the following criteria: sex, age (±1 year), and self-reported amount of alcohol consumed in the last 30 days (±2 standard units per day).

### 2.3. Symptom-Triggered Treatment

The HAES is a validated scale for symptom-triggered alcohol withdrawal treatment and consists of six items: (1) history of delirium and/or seizures, (2) heart rate, (3) systolic blood pressure, (4) tremors, (5) sweating, and (6) clinical overall impression. Defined point values are assigned according to the severity of each item. Based on the sum score (0 to 20 points), the severity of the AWS and the current medication needs can be objectively determined (0–3 points = no medication; 4–6 points = 7.5 mg oxazepam; 7–9 points = 15 mg oxazepam). The application of the HAES starts when the BAC has fallen below 1.0‰. During the critical first 24 h, symptoms are assessed by trained nurses every 1–2 h and then every 2–4 h thereafter. Once the physical AWS subsided (a score of ≤3 points at three consecutive assessments), monitoring via the HAES is discontinued.

### 2.4. Data Collection

The demographic and clinical data were extracted from the electronic medical database. The data sets were anonymized and transferred to a statistical software program on an independent computer for further analysis. The sociodemographic characteristics included information on age and sex. The clinical variables included the duration of AUD, the number of standard units of alcohol in the last 30 days, and history of delirium or seizures, as well as comorbid mental disorders, which were assessed during a structured clinical interview at admission, based on the ICD-10 diagnostic criteria. Moreover, BAC at admission and at the onset of the HAES; gamma-glutamyltransferase (GGT) level at admission; duration of the acute detoxification phase and of HAES monitoring; maximal HAES score; total dosage of benzodiazepines; occurrence of delirium or seizures during withdrawal treatment; transfer at a somatic hospital due to acute physical health problems; length of inpatient stay; and type of discontinuation of treatment were recorded. The acute detoxification phase was defined as the time period (in hours) from admission until the last benzodiazepine administration. All benzodiazepines prescribed during the acute detoxification phase, including any rescue medication for the treatment of complications, in both the FDR and the STT groups were converted into diazepam equivalent doses for standardization.

### 2.5. Data Analysis

Data extraction and analysis were carried out between August 2024 and February 2025. Inspection of the data revealed some missing data in single items, which were not imputed. Sample characteristics from the case–control procedure were compared using paired *t*-tests for continuous variables and Fisher’s exact tests for discrete variables. Also, outcome measures were in the first step compared between groups using paired *t*-tests or Fisher’s exact tests, unadjusted for other explanatory variables. Next, the factors associated with the primary outcome measure, i.e., the total dosage of benzodiazepines, were analyzed using a linear mixed model. This model adjusted for several fixed effects, such as the study group, standard units of alcohol, psychiatric comorbidities, treatment discontinuation, the occurrence of any complication during withdrawal, age, and sex. Case–control pairs were included as random effects. Some variables were not included due to multicollinearity (i.e., the duration of AUD, GGT, and BAC at admission). Finally, a similar mixed model was computed for the duration of the acute detoxification phase, with the total dosage of benzodiazepines included as an additional fixed effect. For mixed models, key model assumptions of normality, homoscedasticity, and the independence of residuals were checked. A two-sided *p*-value of less than 0.05 was considered statistically significant. All analyses were performed using R (4.1.0), with the ‘nlme’ package (3.1–152) used for the mixed models.

Inspection of the HoNOS data at admission revealed missing data in single items. None of the cases, however, had a HoNOS rating with missing data in more than three items. We calculated the total HoNOS score by adding up the scores of the 12 items, which provided a total score ranging from 0 to 48. In cases in which HoNOS items were missing, we imputed the missing values.

## 3. Results

### 3.1. Study Sample

A total of 246 patients with AUD were included in the study, with 123 patients treated with STT and 123 matched patients treated with FDR. The sample predominantly consisted of men (76%) and had a median age of 52 years. The patients had an AUD duration of 12 years and an alcohol consumption of about 500 standard units in the past 30 days, which corresponds to about 17 standard units of alcohol per day. With regard to complications in the past, 12 patients (4.9%) had experienced delirium, and 25 patients (10.2%) had experienced epileptic seizures during previous detoxifications. The majority of patients (69.9%) had at least one comorbid mental disorder; the most frequent comorbidities were affective (58.1%) or neurotic, stress-related, and somatoform (12.2%) disorders. The STT and FDR groups did not differ regarding any demographic, clinical, or alcohol-related variables (Table 1).

### 3.2. Comparison of Treatment Outcomes by Treatment Group

Ten patients in the STT group (8.1%) and four patients in the FDR group (3.3%) did not require any benzodiazepines for alcohol detoxification; the difference was not statistically significant. The implementation of the HAES protocol was associated with a significantly lower median total benzodiazepine dosage, converted into diazepam equivalents, of 22.5 mg, compared to 115.0 mg in the FDR group, corresponding to a reduction of more than 80% (Table 2). In addition, the median duration of the acute detoxification phase was significantly shorter in the STT group (48.0 h) compared to the FDR group (201.8 h), corresponding to a reduction of more than 75%. This finding was accompanied by a significantly shorter median length of inpatient stay in the STT group (23.0 days) compared to the FDR group (28.0 days). There were no significant differences in the rates of delirium (STT: 1.6%; FDR: 0%), epileptic seizures (STT: 1.6%; FDR: 0%), transfers to a somatic hospital (STT: 3.2%; FDR: 0%), or premature discontinuation of treatment (STT: 6.5%; FDR: 3.3%).

As shown in Table 1, the STT group had a median HAES monitoring duration of 80.0 h and a median maximum HAES score of 6.0 points. These observations provide additional insights into the treatment process but did not directly impact the primary outcomes of the study.

### 3.3. Predictors of Benzodiazepine Dosage and Duration of Acute Detoxification

Data analysis using linear mixed models revealed that the differences in total benzodiazepine dosage and the duration of the acute detoxification phase between the STT and FDR groups remained highly significant even after adjusting for various explanatory variables, such as age, sex, the number of standard units of alcohol in the past 30 days, comorbid mental disorders, the premature discontinuation of treatment, and any complications during treatment (Table 3). Other significant predictors of total benzodiazepine dosage included the number of standard units of alcohol in the past 30 days, as well as age and sex, each contributing to the variability in benzodiazepine prescription. In detail, higher doses of alcohol, higher age, and being male were associated with a higher total dosage of benzodiazepines. On the other hand, premature discontinuation of treatment was associated with a shorter duration of the acute detoxification phase, and the total dosage of benzodiazepines was identified as a significant predictor of a prolonged detoxification phase. Both comorbid mental disorders and the occurrence of any complication during treatment did not affect the total benzodiazepine dosage or the duration of the acute detoxification phase.

A coefficient of determination of R^2^ = 0.47 for the total benzodiazepine dosage indicates only a moderate level of correlation between the independent and dependent variables in the model. It can be assumed that unmeasured patient factors may account for the unexplained variability. On the other hand, A coefficient of determination of R^2^ = 0.76 for the duration of the acute detoxification phase indicates a relatively strong relationship between the variables in the model.

## 4. Discussion

The present study aimed to evaluate the effectiveness and safety of STT for inpatient alcohol detoxification following the implementation of the HAES protocol, in comparison to the pre-implementation of the FDR approach. To the best of our knowledge, this is the first study on this issue using a case–control design with a relatively large sample size and carefully matched patients in a clinical setting at a specialized treatment center for addictive disorders, thereby enhancing baseline comparability and strengthening the internal validity of the findings.

With regard to the primary outcome measures, the total benzodiazepine dosage for alcohol detoxification was reduced by more than 80%, and the duration of the acute detoxification phase was reduced by more than 75%, both in the STT group and compared to the FDR group. The results indicate that STT may prevent overtreatment during alcohol detoxification as patients only received the necessary dosages of benzodiazepines based on the individual severity of withdrawal symptoms. The shorter duration of the acute detoxification phase in the STT group may have allowed for an earlier integration of the patients into psychoeducational and psychosocial interventions. Additionally, it might have been responsible for the reduction in the overall length of inpatient stay by day five.

Similar results were obtained by several previous retrospective cohort studies in specialized psychiatric settings, as well as in emergency and general medical departments [14,15,16,17], and by some randomized controlled trials [18], all of them reporting reductions in benzodiazepine prescription and the duration of detoxification of up to 80%. The demographic and clinical characteristics were largely comparable across studies, particularly with regard to age, sex distribution, duration of AUD, current alcohol consumption, and history of complications, thereby ensuring sufficient generalizability. However, there were some differences in the study designs between the various studies, which need to be considered when interpreting the results of this study. In particular, most randomized controlled trials involved rather low-risk populations with a high proportion (up to 60%) of patients who did not require any benzodiazepines in the STT, thereby limiting meaningful comparisons [18].

Compared to these trials, the population of this study included a relatively high proportion of patients with moderate to severe AWS requiring benzodiazepine prescriptions, with only 8% of the patients in the STT not requiring any pharmacological intervention. Moreover, a prevalence rate of comorbid mental disorders of about 70% was rather high in this study, while other studies have explicitly excluded patients with major psychiatric disorders [19]. Finally, most previous studies have used the CIWA-Ar scale for the management of alcohol detoxification, in comparison to the present study, which applied the HAES protocol, a simple and quick assessment tool used to evaluate the severity of AWS with only six items [23]. Taken together, STT was highly effective even in patients with severe AWS and/or comorbid mental disorders, indicating that the HAES protocol can be efficiently applied even in heterogeneous clinical samples.

Despite the considerably lower doses of benzodiazepines, however, the rates of complications, such as delirium and epileptic seizures during treatment, transfers to a somatic hospital, and premature discontinuation of treatment, were rather rare and did not differ significantly between the two treatment settings. Moreover, the occurrence of any complications did not affect the total benzodiazepine dosage or the duration of the acute detoxification phase. This finding is in accordance with most previous studies [14,15,16,17] and underlines that STT appears to be as safe as FDR. However, it should be mentioned that all four patients who experienced delirium or epileptic seizures during treatment were in the STT group, whereas no complications were observed in the FDR group. Therefore, it cannot completely be excluded that the occurrence of these events might have been due to insufficient doses of benzodiazepines.

Comorbid mental disorders were highly prevalent among the study population, particularly affective and stress-related disorders, with no significant differences in the prevalence rates between the STT and FDR groups. Although one might expect that comorbid mental disorders would have the potential to complicate the course of alcohol detoxification, they did not affect the total benzodiazepine dosage or the duration of the acute detoxification phase in this study. This finding is broadly consistent with a previous study that also showed that STT remained effective even in the presence of mental comorbidities [15], suggesting that the HAES protocol may be robust across a range of psychiatric conditions. However, it cannot be excluded that potential medication prescribed for the treatment of comorbid mental disorders might have affected the primary withdrawal outcomes.

Further analysis of the potential predictors of total benzodiazepine dosage and the duration of acute detoxification revealed that higher alcohol intake and higher age were significantly associated with a higher total dosage of benzodiazepines. This finding is not surprising, as patients with excessive alcohol use, as well as elderly patients, were repeatedly reported to be at a higher risk of the development of severe AWS, whose management typically requires more intensive pharmacotherapy [26,27]. The total dosage of benzodiazepines was, in turn, a significant predictor of a prolonged detoxification phase, which is necessary in order to safely taper off the medication. As expected, male patients required significantly higher total dosages of benzodiazepines than female patients. This finding is in line with various studies that have consistently reported that men were more likely than women to develop severe AWS, including delirium and seizures, and, therefore, received benzodiazepines at higher dosages [28].

The study has several limitations. (1) The retrospective study design does not offer the same level of evidence as a randomized controlled trial. However, group comparability was strengthened by using a case–control approach with a relatively large number of carefully matched patients. (2) Although patients were matched on sex, age, and alcohol intake, residual confounding from unmeasured variables may persist. (3) As the study relied on electronic medical records, incomplete or inconsistent documentation may have affected the accuracy of the extracted data. (4) The use of the HAES for STT does not allow for direct comparisons with previous studies that used the CIWA-Ar scale. (5) Although our sample included a substantial proportion of patients with comorbid mental disorders, categorized under affective, stress-related, or “other” disorders, these classifications were relatively broad, thereby limiting our ability to assess the impact of specific severe disorders, such as psychosis or personality disorders. (6) All benzodiazepines were converted into diazepam equivalents for consistency; however, such conversions may introduce minor inaccuracies due to pharmacokinetic and pharmacodynamic variability among different benzodiazepines and individual patients. (7) This study was conducted at a single specialized psychiatric treatment center, potentially limiting the generalizability to other healthcare settings, especially those with fewer resources or different patient demographics. (8) Although our results indicate superior short-term outcomes for the STT group, we did not track long-term parameters such as relapse rates, sustained abstinence, or readmission risks. Future research should incorporate extended follow-up to assess whether reduced benzodiazepine use during detoxification has a positive impact on long-term recovery.

## 5. Conclusions

This study provides evidence that STT using the HAES protocol appears to be as effective and safe as traditional FDR for the management of alcohol detoxification in a specialized inpatient setting. In particular, STT significantly reduced benzodiazepine prescription, the acute detoxification period, and the length of inpatient stay. It can be speculated that these outcomes are associated with minimized unnecessary pharmacological exposure and reduced healthcare costs. Additionally, STT possibly facilitates the earlier integration of patients into psychoeducational and psychosocial interventions due to reduced withdrawal burden and a shorter detoxification phase. On the other hand, the rates of complications during treatment did not differ between STT and FDR. Further prospective studies are required to validate these findings and assess long-term outcomes.

## Figures and Tables

**Table 1:** Demographic and clinical characteristics of the two study groups (total N = 246).

	All (N = 246)		STT Group (N = 123)		FDR Group (N = 123)		*p* ^1^
**Demographic characteristics**							
Age (years), median, IQR	52.0	43.0–56.0	52.0	43.5–56.0	51.0	43.0–56.0	n.s.
Sex (male), N, %	188	76.4	94	76.4	94	76.4	n.s.
**Clinical characteristics**							
Duration of AUD (years), median, IQR	12.0	6.0–20.0	12.0	6.0–20.0	12.0	6.0–20.0	n.s.
Standard units of alcohol in the past 30 days, median, IQR	499.8	390.0–799.8	499.8	395.0–804.9	500.0	375.0–789.9	n.s.
History of delirium (yes), N, %	12	4.9	7	5.7	5	4.1	n.s.
History of epileptic seizure (yes), N, %	25	10.2	14	11.4	11	8.9	n.s.
Comorbid mental disorder (yes), N, % – F3 affective disorders – F4 neurotic, stress-related, and somatoform disorders – Other diagnoses	172 143 30 52	69.9 58.1 12.2 21.1	87 71 12 22	70.7 57.7 9.8 17.9	85 72 18 30	69.1 58.5 14.6 24.4	n.s. n.s. n.s. n.s.
GGT at admission (IU/mL), median, IQR	131.0	63.5–420.0	130.0	68.0–287.0	151.0	62.3–456.0	n.s.
BAC at admission (‰), median, IQR	1.2	0.1–1.8	1.3	0.2–1.9	1.2	0.1–1.7	n.s.
BAC at HAES onset (‰), median, IQR			0.8	0.0–1.7			
Maximum HAES score, median, IQR			6.0	5.0–8.0			
Duration of HAES monitoring (hours), median, IQR			80.0	56.9–115.9			

^1^ Paired *t*-tests for continuous data; Fisher’s exact test for categorical data. STT, symptom-triggered therapy; FDR, fixed-dose regimen; IQR, interquartile range; AUD, alcohol use disorder; GGT, gamma-glutamyltransferase; BAC, breath alcohol concentration; HAES, Hamburg Alcohol Withdrawal Scale.

**Table 2:** Treatment outcome parameters and adverse events in the two study groups (total N = 246).

	All (N = 246)		STT Group (N = 123)		FDR Group (N = 123)		*p* ^1^
Total benzodiazepine dosage ^2^ (mg), median, IQR	58.8	20.0–120.0	22.5	8.8–40.0	115.0	83.8–160.0	<0.001
Duration of acute detoxification phase (hours), median, IQR	110.0	38.5–202.0	48.0	22.8–95.6	201.8	152.3–249.8	<0.001
Length of inpatient stay (days), median, IQR	27.0	14.0–37.0	23.0	10.5–35.5	28.0	21.0–40.0	0.003
Delirium during treatment (yes), N, %	2	0.8	2	1.6	0	0	n.s.
Epileptic seizure during treatment (yes), N, %	2	0.8	2	1.6	0	0	n.s.
Transfer to somatic hospital during treatment (yes), N, %	4	1.6	4	3.2	0	0	n.s.
Premature discontinuation of treatment (yes), N, %	12	4.9	8	6.5	4	3.3	n.s.

^1^ Paired *t*-tests for continuous data; Fisher’s exact test for categorical data; all values two-tailed. ^2^ Diazepam equivalents. STT, symptom-triggered therapy; FDR, fixed-dose regimen; IQR, interquartile range.

**Table 3:** Linear mixed models for total benzodiazepine dosage and duration of acute detoxification phase.

	Model for Total Benzodiazepine Dosage			Model for Duration of Acute Detoxification Phase		
Predictor	*β* Coefficient	95% CI	*p*	*β* Coefficient	95% CI	*p*
(intercept)	20.95	−26.99, 68.89	0.389	110.76	63.73, 157.79	<0.001
STT group	−98.42	−112.65, −84.2	<0.001	−31.80	−50.04, −13.56	<0.001
Standard units of alcohol in the past 30 days	0.03	0.01, 0.06	<0.005	−0.01	−0.03, 0.01	0.449
Comorbid mental disorder	11.63	−3.97, 27.23	0.142	−11.07	−26.13, 3.99	0.148
Premature discontinuation of treatment	−5.46	−38.7, 27.78	0.746	−37.27	−70.54, −3.99	<0.05
Any complication during treatment	21.89	−24.6, 68.38	0.353	11.51	−33.32, 56.35	0.612
Age	1.19	0.40, 1.98	<0.005	−0.55	−1.34, 0.24	0.170
Sex (male)	25.08	−41.88, −8.28	<0.005	−10.39	−6.38, 27.17	0.222
Total benzodiazepine dosage	--	--	--	1.12	0.99, 1.24	<0.001
	marginal R^2:^ 0.47 conditional R^2:^ 0.47			marginal R^2:^ 0.76 conditional R^2:^ 0.77		

CI, confidence interval; STT, symptom-triggered therapy.

## Data Availability

The raw data supporting the conclusions of this article will be made available by the authors upon request.
